# RNA tomography reveals spatial gene expression maps of *Arabidopsis thaliana* roots infected with *Heterodera schachtii*

**DOI:** 10.1111/nph.70674

**Published:** 2025-10-17

**Authors:** Anna Pijnacker, Yuhao Wang, Jaap‐Jan Willig, Jonas Mars, Steffen Werner, Kelvin Adema, Geert Smant, Hendrik C. Korswagen, Jose L. Lozano‐Torres

**Affiliations:** ^1^ Laboratory of Nematology Wageningen University & Research Wageningen 6708 PB the Netherlands; ^2^ Agrosystems Research Wageningen University & Research Wageningen 6708 PB the Netherlands; ^3^ Hubrecht Institute Utrecht 3584 CT the Netherlands; ^4^ Experimental Zoology Wageningen University & Research Wageningen 6708 WD the Netherlands; ^5^ Laboratory of Cell and Developmental Biology Wageningen University & Research Wageningen 6708 PB the Netherlands; ^6^ Department of Biology, Institute of Biodynamics and Biocomplexity, Developmental Biology Utrecht University Utrecht 3584 CH the Netherlands

**Keywords:** Arabidopsis, cyst nematode, nematode infection, plants, RNA tomography, spatial gene expression maps, spatial transcriptomics

## Abstract

- Plant‐parasitic cyst nematodes, such as *Heterodera schachtii*, cause substantial crop losses world‐wide and induce specialized feeding structures in host roots, yet the molecular mechanisms underlying feeding structure initiation and development remain poorly understood.
- We introduce RNA tomography for plants, a powerful untargeted spatial transcriptomics technology that allows studying gene expression at high spatial resolution. We applied RNA tomography to Arabidopsis (*Arabidopsis thaliana*) roots infected with *H. schachtii*, capturing 96 consecutive cross sections of 20 micrometers at 1‐ and 2‐d post inoculation (dpi).
- We identified the location of the nematode's pharyngeal glands, the organs where most effectors are produced, using marker genes, and discovered multiple uncharacterized *H. schachtii* genes expressed in the same region. Additionally, we mapped the Arabidopsis spatial gene expression response upon nematode infection, revealing that some genes are expressed in a specific section.
- Our findings provide novel insights into early nematode parasitism. RNA tomography offers a powerful new approach to understanding plant cellular organization and interactions under various conditions, including development and responses to biotic and abiotic stresses.

## Introduction

Plants are complex organisms with multiple cell types organized into tissues and organs. These cells communicate to coordinate plant development and responses to environmental stimuli, such as pathogen infection (Tabassum & Blilou, 2022). Understanding gene expression variations between cells is crucial for unraveling the regulatory gene networks that control these processes. Spatial gene expression patterns are particularly important for unraveling localized processes, such as the response upon infection with the economically important sedentary plant‐parasitic cyst nematodes (Jones *et al*., 2013).

Infective juveniles (J2) of cyst nematodes penetrate the roots and migrate intracellularly toward the vascular cylinder (Golinowski *et al*., 1996), injecting effector proteins via a needle‐like stylet (Vanholme *et al*., 2007; Hewezi *et al*., 2008; Lee *et al*., 2011; Mitchum *et al*., 2013). After migration, cyst nematodes initiate a permanent feeding structure, the syncytium, from a single vascular cell (Wyss, 1992; Golinowski *et al*., 1996; Sobczak *et al*., 1997). The syncytium expands over weeks through partial cell‐wall dissolution and protoplast fusion of hundreds of adjacent cells (Golinowski *et al*., 1996). However, the molecular mechanisms underlying the initiation and expansion of nematode‐induced feeding structures are poorly understood, as is the heterogeneity within these structures, such as the existence of hundreds of nuclei within a single cytoplasm. This is largely due to a lack of methods for mapping spatial transcriptomic variation within and at the perimeter of nematode‐induced feeding structures (Szakasits *et al*., 2009; Barcala *et al*., 2010; Damiani *et al*., 2012; Matuszkiewicz *et al*., 2018).

To date, traditional transcriptomics, such as RNA microarrays (Puthoff *et al*., 2003; Khan *et al*., 2004; Uehara *et al*., 2010) and RNA sequencing (Hosseini & Matthews, 2014; Piya *et al*., 2017), have been used to study plant responses to nematode infections. However, these approaches analyze whole‐root systems of cyst‐nematode‐infected plants and hence do not differentiate gene expression in specialized feeding structures from surrounding plant cells. Given that feeding structures represent a small fraction of the root, transcriptional changes important for feeding structure formation might remain undetected. Methods like laser capture microdissection (Ithal *et al*., 2007) and microaspiration (Szakasits *et al*., 2009), isolate RNA specifically from syncytial elements induced by *Heterodera spp*. (Matuszkiewicz *et al*., 2018), but require pooling of clearly visible feeding structures of multiple nematodes, that is, 3 d post inoculation (dpi). Due to the asynchronous nature of nematode infections in terms of feeding structure initiation, these pooled structures encompass different developmental stages of feeding structures. More recently, RNA sequencing was applied to compare samples of pooled dissected root segments containing nematodes with similar pools of adjacent uninfected root tissue (Siddique *et al*., 2022). Although this approach differentiates between infected and uninfected tissue, it lacks the resolution to capture the dynamics of the transcriptome across infected tissue.

To study gene expression changes in and around the developing nematode‐induced feeding structures at the multi‐cellular level, spatial transcriptomics is required. Two main spatial transcriptomics approaches are commonly used in plants: *in situ* capture sequencing‐based methods and imaging‐based methods, relying on fluorescence *in situ* hybridization (FISH) (Cheng *et al*., 2023; Yin *et al*., 2023). In plants, the cellular resolution provided by *in situ* capturing sequencing has been crucial for identifying cell types (Xia *et al*., 2022), cell heterogeneity (Liu *et al*., 2022), and cellular organization (Liu *et al*., 2023), while FISH‐based spatial transcriptomics methods have revealed cell‐type markers in roots (Nobori *et al*., 2023), leaf venation patterns (Perico *et al*., 2024), and spatial expression of pathogen‐responsive genes (Nobori *et al*., 2025). *In situ* capture sequencing‐based methods enable the transcriptome‐wide quantification and localization of gene expression with high throughput (Giacomello *et al*., 2017; Giolai *et al*., 2019; Xia *et al*., 2022). By contrast, FISH‐based techniques demand prior knowledge of target genes and are limited in throughput by the number of fluorescent probes that can be used simultaneously (Veselinyová *et al*., 2021). Both approaches rely on microscope slides, which restricts the tissue capture area. As a result, analyzing the transcriptional variation in larger structures requires multiple slides, increasing costs and making spatial transcriptomics of large structures unfeasible for most groups (Nobori *et al*., 2023).

Here, we introduce RNA tomography, or Tomo‐seq, a spatial transcriptomics technology that maps gene expression patterns at high resolution by combining serial tissue sectioning with RNA sequencing without the use of a microscope (Junker *et al*., 2014; Holler & Junker, 2019). After cryo‐sectioning a sample along a specific axis, mRNA is labeled with section‐specific barcoded primers and unique molecular identifiers (UMIs). Barcodes retain spatial information, while UMIs help distinguish individual molecules and mitigate artifacts resulting from PCR‐based amplification (Hashimshony *et al*., 2012). This amplification step allows researchers to begin with minimal input material. RNA tomography has provided high‐resolution spatial gene expression maps in several animals (Junker *et al*., 2014; Wu *et al*., 2016; Ebbing *et al*., 2018; Yvernogeau *et al*., 2020; Mayeur *et al*., 2021; Schild *et al*., 2021) but has not yet been applied to plants or to two species simultaneously.

We applied RNA tomography to Arabidopsis (*Arabidopsis thaliana*) roots at 1 and 2 dpi with the beet cyst nematode *Heterodera schachtii* to capture key transitions in feeding structure initiation and expansion (Golinowski *et al*., 1996). Our results provide high‐resolution spatial gene expression maps of nematode‐infected Arabidopsis roots and reveal gene expression within the nematode itself. We bioinformatically identified the position of the nematode within the root and studied Arabidopsis gene expression up to 400 micrometers from the nematode's anterior side. Through RNA tomography, we can greatly enhance our understanding of the spatial organization of plant cells and their interactions under various conditions, including plant development and responses to biotic and abiotic stresses.

## Materials and Methods

### RoPod preparation

To simplify the screening for nematode‐infected Arabidopsis roots and reduce the number of steps that seedlings must be transferred, RoPod microscopic chambers were used (Guichard *et al*., 2024). Eighteen‐well RoPods were 3D‐printed with a Prusa MKS3+ printer using white PLA (EAN 6922572219038; Esun, Shenzhen, China) and 24 × 60 mm #1.5 coverslips (cat # 15 165 452; Epredia, Breda, the Netherlands), according to the protocol of Guichard *et al*. (2024). RoPods were carefully washed with water and soap, and sterilized using UV light (Philips tuv 30 W/G30T8) treatment for at least 1 h before use. Subsequently, 3 ml of sterile modified Knop medium (Sijmons *et al*., 1991) was pipetted into the RoPod. After the medium solidified, a strip of the medium was removed using a sterile toothpick.

### Plant culturing conditions

Seeds of the double auxin and cytokinin reporter DR5:TCSn in *Arabidopsis thaliana* Col‐0 background (kindly provided by Dolf Weijers from the Laboratory of Biochemistry, Wageningen University and Research, the Netherlands) were used to localize auxin and cytokinin expression using confocal microscopy. The confocal data is not shown in this paper. Seeds were stratified for 3 d at 4°C in the dark and vapor sterilized in an exicator with a final concentration of 50 g l^−1^ sodium hypochlorite (2102 949; Boom, Meppel, the Netherlands) and 25% hydrochloric acid (7647‐0‐10; Merck, Darmstadt, Germany) for 3 h. After sterilization, seeds were sown in the arch of the RoPod at the edge of the medium using sterile toothpicks. The RoPods were placed in a square Petri dish (120 × 120 mm) sealed with household foil and vertically placed in a growth cabinet at 21°C with 16 h light : 8 h dark cycles using cool white light (MASTER TL‐D Super 80 36 W1840; Philips, Eindhoven, the Netherlands).

### Hatching and sterilization of *Heterodera schachtii*

*Heterodera schachtii* cysts (Woensdrecht population from IRS, the Netherlands) were isolated from the sand of *Brassica oleracea* infected plants as previously done by Baum *et al*. (2000). Hatching and sterilization of *H. schachtii* J2 were performed as previously described (Willig *et al*., 2022). Four‐day‐old Arabidopsis seedlings were inoculated with 20 *H. schachtii* J2 per 3 seedlings, allowing the J2 to move between all 18 seedlings within the RoPod.

### Sample preparation and sectioning

Samples for RNA tomography were taken at 1 and 2 dpi. Seedlings were screened for a nematode‐infection using a Zeiss Axio imager M2 light microscope with a ×10 objective and ×20 magnification. Infected seedlings in which the position of a single nematode within the root was clearly visible were selected and imaged with an AxioCam MRc5 camera (426560‐9080‐000; Zeiss) using the ZEN software (2009; Zeiss). Subsequently, infected seedlings were transferred to a disposable mold (720‐0820; VWR) filled with a layer of tissue‐freezing medium (14 020 108 926; Leica, Richmond, IL, USA). Using an Olympus SZX10 binocular with a ×1.5 objective and ×2.5 magnification, a clump of Affi‐Gel Blue Gel beads (1537 301; Bio‐Rad, Dreiech, Germany) was placed near the nematode glands, indicating the starting point for sectioning. Pictures of the position of the blue beads relative to the sample were taken with an AxioCam 712 color camera (426560‐9080‐000; Zeiss) using the zen software (2009; Zeiss). Samples were flash‐frozen on dry ice within 10 min after transferring the seedling to tissue‐freezing medium. Directly after freezing, the orientation of the root and the location of the nematode were marked on the mold and frozen sample using a marker. The frozen samples were stored at −80°C until use. Excess tissue‐freezing medium was removed before sectioning in cryosections of 20 μm using a SLEE MEV cryostat set at −18°C, maximally 24 h before RNA amplification. The sections were collected using tweezers in a pre‐cooled hard‐shell 96‐wells plate filled with 40 μl mineral oil (M8410; Sigma‐Aldrich) and 7.5 ng μl^−1^ primer containing a T7 promoter, Illumina 5′adapter, 6 bp UMI, an 8 bp section‐specific barcode and a poly‐T‐sequence (Schild *et al*., 2021). The T7 promoter in the Cel‐Seq2‐based primers (Hashimshony *et al*., 2016) facilitates *in vitro* transcription but is not involved in the sequencing reaction. The UMI distinguishes molecules from each other, while the barcodes contribute to the spatial aspect of the Illumina sequencing library. Primers were synthesized by Integrated DNA Technologies (Leuven, Belgium).

### RNA amplification, cDNA synthesis, and Illumina library preparation

To each tissue section, 0.1 μl of 10 mM dNTPs (Promega) and 0.2 μl (1 : 500 000 diluted) ERCC spike‐ins were added using the Nanodrop II robot (GC Biotech, Waddinxveen, the Netherlands). The plate was heated to 65°C for 5 min to release the RNA and spun down in a pre‐chilled centrifuge at 20 000 ***g*** for 10 s. Next, a mix containing 0.05 μl nuclease‐free water, 0.4 μl First Strand Buffer (5×), 0.2 μl DTT (0.1 M), 0.1 μl RNAse OUT (40 U μl^−1^), and 0.1 μl SuperScript II (200 U μl^−1^) was prepared, and 0.75 μl of this mix was added to each well using the Nanodrop II for reverse transcription before incubation for 1 h at 42°C, 5 min at 4°C, and 10 min at 70°C. To generate a second‐strand cDNA, a mix consisting of 7 μl nuclease‐free water, 2.5 μl Second Strand Buffer (5×), 0.25 μl dNTPs (10 mM), 0.35 μl *Escherichia coli* DNA ligase (10 U μl^−1^), 0.09 μl *E. coli* DNA polymerase (10 U μl^−1^), and 0.09 μl *E. coli* RNAse H (2 U μl^−1^) was prepared, and 10 μl of the mix was added to each well, followed by a 2 h incubation step at 16°C. NucleoMag NGS Clean‐up and Size Select beads (LOT 102228; Machery‐Nagel, Düren, Germany) were used to purify the cDNA (slightly modified from Schild *et al*. (2021)). Next, the amplified cDNA was transcribed into RNA using the MEGAScript T7 transcription kit (CAT#AM1334; Ambion, Carlsbad, CA, USA), and the RNA was fragmented by incubating the sample with aRNA‐fragmentation buffer at 94°C for 1.5 min. The RNA was then cleaned up using RNA MagClean beads (C‐100; Aline Biosciences, Nashua, NH, USA) before preparing the DNA library, following the protocol of Schild *et al*. (2021).

### Read mapping

The libraries were sequenced twice on the Illumina NextSeq 2000 platform using P3 reagents (100 cycles, 240 Gb) at Leiden University (The Netherlands) to achieve sufficient sequencing depth. fastq files from both runs, containing paired‐end 100 base pair sequencing reads, were merged. Read 1 was used for transcript counting based on the UMI and section‐specific barcode, while Read 2 contained the gene‐specific data. To process the raw fastq data, we used the SingleCellMultiOmics (scmo v.0.1.40) (‘GitHub – BuysDB/SingleCellMultiOmics: Tools which deal with multiple single cell measurements’, 2024) pipeline. First, the UMI and barcodes from Read 1 were demultiplexed and added to the head of Read 2 by using demux.py (scmo v.0.1.40). The demultiplexed reads were then mapped to the Arabidopsis genome (Araport11) (Cheng *et al*., 2017) using star (v.2.7.11) (Dobin *et al*., 2012). Mapped reads were assigned to genes with featurecounts (v.2.0.8) (Liao *et al*., 2014), sorted with samtools (v.1.6) (Li *et al*., 2009), and assigned to the barcode molecules using bamtagmultiome.py (scmo v.0.1.40). We then counted the duplicated and non‐duplicated UMIs per gene per section using bamToCountTable.py (scmo v.0.1.40). In the second step, the demultiplexed reads were mapped to the *H. schachtii* genome (Bonn population; Siddique *et al*. 2022) using STAR, following the same procedure as described in the first step. The non‐duplicated UMI counts from both plant and nematode genomes were used for downstream analyses.

### Bioinformatic analyses

Transcript counts based on non‐duplicated UMI were analyzed using R (v.4.3.2). To assess the quality of sections, we plotted the cumulative distribution of the sum of Arabidopsis and *H. schachtii* genes per section. To assess the quality of sections, the number of Arabidopsis and *H. schachtii* genes detected per section was summed and then sorted in ascending order. The cumulative distribution of these gene counts was plotted and used to identify the largest discontinuity between neighboring sections. This discontinuity indicates a superposition of two distinct behavioral regimes, corresponding to low‐ and high‐quality sections. The largest discontinuity occurred at 300 total genes per section, thus sections with fewer than 300 total genes were considered low quality and excluded from further analysis (Supporting Information Fig. S1a,b). The remaining gene‐section matrix was normalized using SCTransform from the seurat package (v.5.2.1) (Hao *et al*., 2023), retaining sections with at least 300 detected genes in at least one section. The number of Arabidopsis and *H. schachtii* genes across sections was compared using ggplot2 (v.3.5.1) (Wickham, 2016) before and after the filtering and normalization. To identify the location of a nematode, we plotted the cumulative distribution of detected *H. schachtii* genes per section, revealing a that the largest discontinuity between neighboring sections occurred at 75 *H. schachtii* genes (Fig. S1c,d). The consecutive sections containing > 75 *H. schachtii* genes were defined as nematode‐containing regions because we assume that a nematode spans multiple consecutive sections. For generating spatial gene expression maps, we used the complexheatmap package (v.2.20.0) with average Euclidean clustering (Gu *et al*., 2016). The optimal number of clusters was determined using the elbow method (Syakur *et al*., 2018), which plots the within‐cluster sum of squares (WCSS) for cluster counts ranging from 1 to 15. The second derivative of WCSS was calculated to identify the point of maximum curvature, indicating the optimal number of clusters for generating spatial gene expression maps.

For *H. schachtii*, we constructed the spatial heatmaps with all detected genes. Furthermore, we selected genes known to be expressed in the dorsal and subventral glands based on published literature (Molloy *et al*., 2024), and visualized their expression in heatmaps. We manually compared the gene IDs expressed in either gland for the 1 and 2 dpi samples and visualized this comparison in a Venn diagram and annotated the genes using the effectorome (Molloy *et al*., 2024) and eggnog‐mapper (v.2) with default settings (Cantalapiedra *et al*., 2021). Additionally, we selected *H. schachtii* gene 21 727 for the 1 dpi sample and *H. schachtii* gene 9937 for the 2 dpi sample, as marker genes for the subventral glands and dorsal gland, respectively. We searched for a maximum of 200 genes with a Spearman correlation higher than 0.9 based on the expression pattern of the selected marker genes and visualized their expression profiles in heatmaps. To investigate sequence similarity, we blasted the protein sequences of *H. schachtii* genes expressed outside the nematode‐containing region, in at least one or two sections, against Arabidopsis proteins.

For analyzing Arabidopsis, we selected the 10 000 most highly variable genes with seurat (v.5.2.1) (Hao *et al*., 2023) for each sample, including both Arabidopsis and *H. schachtii* genes, as we expect spatial variation in gene expression due to feeding structure formation. The 9732 highly variable Arabidopsis genes from 1 dpi and 9053 genes from 2 dpi were visualized using the complexheatmap (v.2.20.0) (Gu *et al*., 2016). The optimal number of clusters was determined using the elbow method and its second derivative, as described above for selecting the appropriate number of clusters for the heatmap visualizing *H. schachtii* genes. The mean expression of genes per cluster was plotted in red on the left of the heatmap. Gene ontology enrichment analysis was performed using shinygo (v.0.82, accessed on 12 and 13 March 2025) (Ge *et al*., 2019) for clusters with high or altered expression in or near the nematode region, where we expect the feeding structure to develop. *P*‐values were calculated using the hypergeometric test, and false discovery rates (FDRs; cutoff 0.05) were computed via the Benjamini–Hochberg method to correct for multiple testing. Fold enrichment was defined as the percentage of genes in our list for a given pathway divided by the corresponding percentage in background Arabidopsis genes. Pathways to be shown were set at 20 (Size Min = 2 and Max = 5000) and selected the top 10.

## Results

### RNA tomography of nematode‐infected Arabidopsis roots captures the spatial gene expression profiles of both species simultaneously

**Fig. 1:**
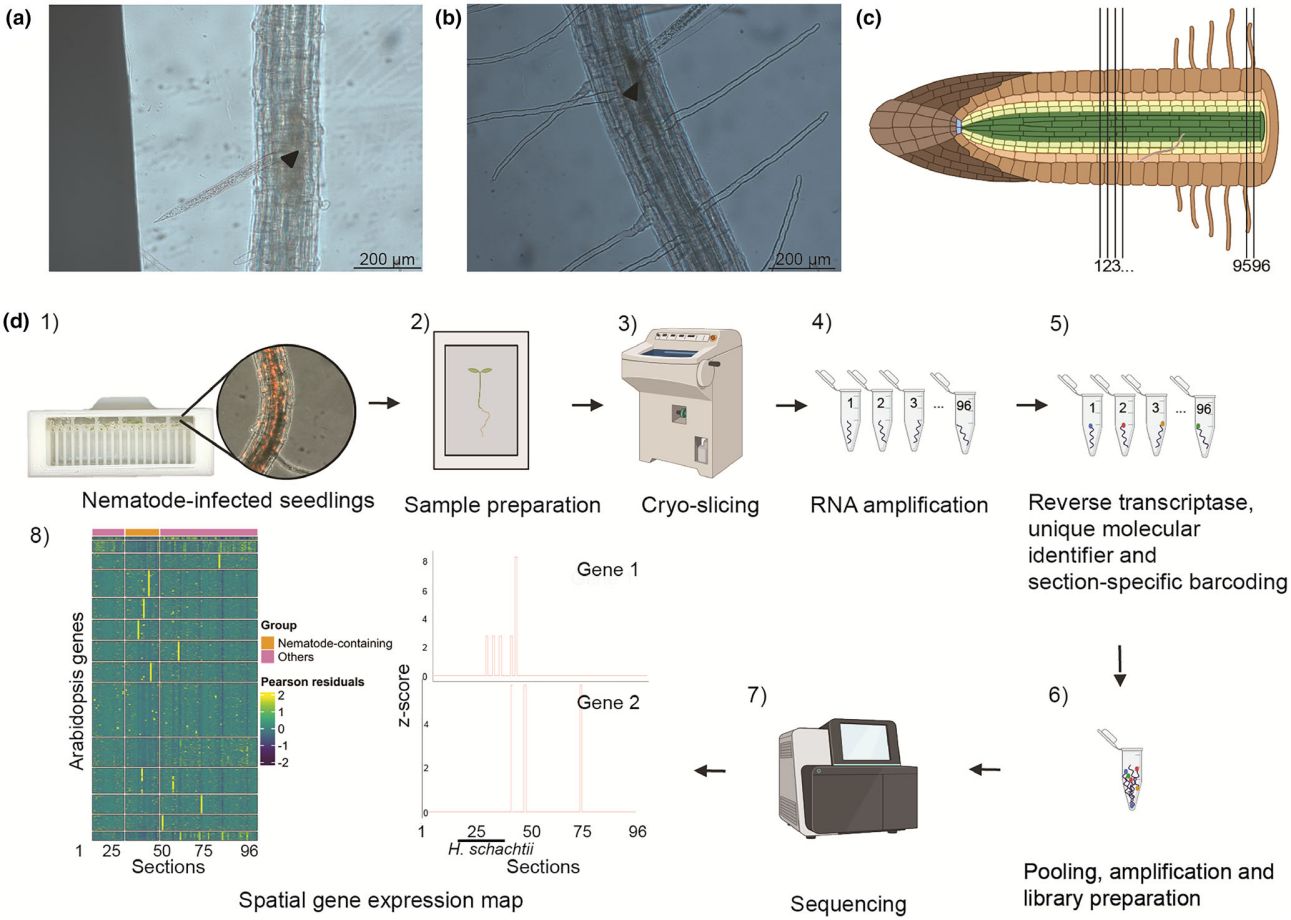
Overview of the RNA tomography sample preparation and workflow. This figure provides an overview of the experimental workflow used for RNA tomography, from sample preparation to analysis. (a, b) Microscopic image of the feeding structure induced by *Heterodera schachtii*, 1 (a) and 2 (b) days post inoculation (dpi), ×20 magnification. The arrowhead indicates the position of the nematode glands. (c) Frozen samples are sectioned into 96 tissue sections, starting near the nematode's anterior end along the plant root. (d) (1) Four‐day‐old *Arabidopsis thaliana* roots were infected with second‐stage *H. schachtii* juveniles in RoPod microscopic chambers. (2) At 1 and 2 dpi, the roots were microscopically screened for nematode infections, imaged, and infected seedlings were transferred to a mold filled with tissue‐freezing medium. (3) Using a cryostat, the sample was sectioned into 96 sections of 20 μm each, starting near the nematode's anterior end. Each section was individually collected. (4) RNA was amplified from each individual section. (5) cDNA was synthesized using section‐specific barcoded primers containing a unique molecular identifier (UMI) to label RNA molecules. (6) Barcoding allowed pooling all sections of one sample together for RNA amplification. Subsequently, the RNA was amplified by *in vitro* transcription, and sequencing libraries were prepared. (7) Libraries were sequenced on the Illumina NextSeq2000. (8) The reads obtained from the paired‐end sequencing analysis were mapped to both reference genomes of Arabidopsis and *H. schachtii* to provide detailed spatial gene expression maps, with the 96 tissue sections presented on the *x*‐axis and the presence of nematode mRNA indicated with a black horizontal line and the text ‘*H. schachtii’*. In the heatmap, the top 1000 most expressed Arabidopsis genes are displayed on the *y*‐axis. The *y*‐axis in the gene expression plots contains *z*‐scores, gene 1 is a gene known to be expressed in the *H. schachtii* glands, and gene 2 is an Arabidopsis gene locally expressed near the nematode gland. (c, d) (2–7) were created in BioRender (BioRender.com/a4f1ncp).

To capture the spatial variation of gene expression at different time points during the early developmental stages of cyst nematode‐induced feeding structures in plant roots, we adapted the RNA tomography protocol used for *C. elegans* for application in plants (Schild *et al*., 2021). Hereto, we inoculated 4‐d‐old Arabidopsis seedlings with infective juveniles of *H. schachtii*. We collected nematode‐infected roots at 1 and 2 dpi (Fig. 1a,b) when feeding structure initiation and the early stages of expansion take place (Golinowski *et al*., 1996). Frozen roots with feeding structures were subsequently sectioned into 96 sections, starting near the anterior pharyngeal gland region of the nematode along the longitudinal axis of the roots into the plant tissue at increasing distance from the nematode (Fig. 1c). Since the nematode head could be oriented toward either end of the plant, the sectioning was done acropetally or basipetally, meaning the head could be located near section 1 or 96, respectively. Each section was incubated with an adapted version of the CelSeq2 primer, to distinguish mRNA molecules from each other, trace back reads from the specific tissue sections and add spatial information to the RNA sequencing library using barcodes (Hashimshony *et al*., 2016). All sequencing reads were either mapped to the Arabidopsis or *H. schachtii* reference genomes (Fig. 1d).

**Fig. 2:**
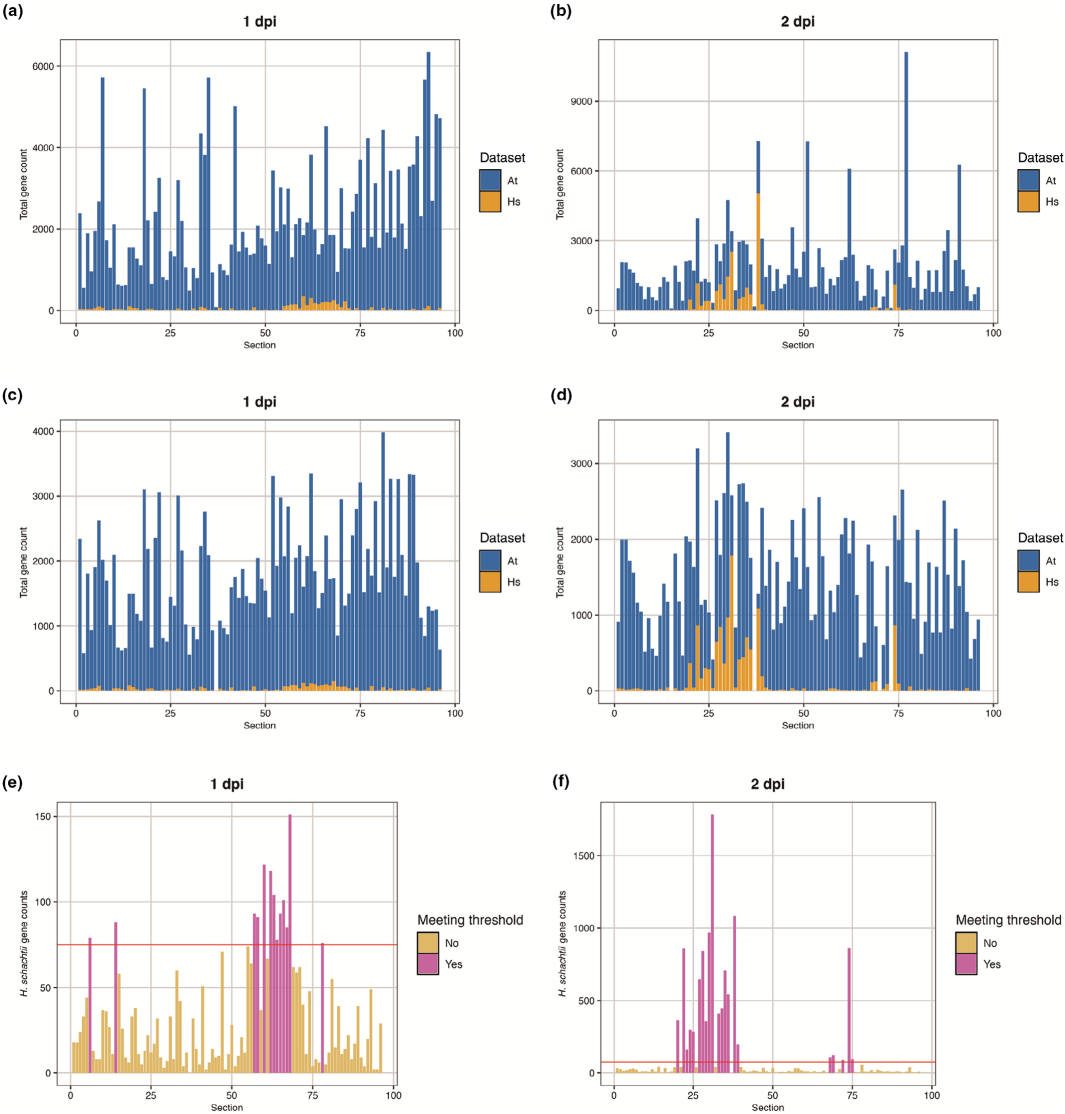
RNA tomography enables the simultaneous detection of genes from both *Arabidopsis thaliana* and the cyst nematode *Heterodera schachtii* in individual sections. The number of detected Arabidopsis and *H. schachtii* gene counts was analyzed across sections at different days post inoculation (dpi). (a, b) Gene counts were plotted across sections for samples collected at 1 (a) and 2 (b) dpi with *H. schachtii*, with Arabidopsis gene counts (At) shown in blue and *H. schachtii* gene counts (Hs) in orange. (c, d) Sections with fewer than 300 total detected genes were filtered out, and the remaining gene counts were normalized using a negative binomial regression model. After filtering and normalization, gene counts were plotted again, revealing that one section was excluded from the 1 dpi sample (c), and four sections were removed from the 2 dpi sample (d). (e, f) To determine the location of *H. schachtii* in the samples, filtered and normalized *H. schachtii* gene counts were plotted for the 1 (e) and 2 dpi (f) samples. Sections containing > 75 detected *H. schachtii* genes were considered nematode‐containing and are shown in pink, while sections below the threshold of 75 genes, indicated by the red line, are represented in orange.

Since RNA tomography has not yet been applied to plants and to two organisms simultaneously, we first assessed whether genes from both the plant and the nematode could be detected within a single section. Therefore, we plotted the number of distinct gene counts per section for both organisms (Fig. 2a,b). Indeed, genes from both the plant and *H. schachtii* were detected across various sections. For instance, in section 60 of the 1 dpi sample, we identified 1499 unique Arabidopsis genes, along with 348 unique *H. schachtii* genes. Similarly, in section 22 of the 2 dpi sample, we detected 2803 unique Arabidopsis genes and 1159 unique *H. schachtii* genes. These findings confirm that RNA tomography can simultaneously capture gene expression from two organisms.

To ensure that observed changes in gene expression are due to biological processes, rather than technical variations, such as differences in sequencing depth or gene counts per section, we applied filtering and normalization. First, we determined a threshold to filter out low‐quality sections by ranking sections based on ascending numbers of detected genes and identifying the largest increase among sections with low numbers of total genes. This increase occurred at 300 total detected genes per section, which we then set as the minimum quality threshold (Fig. S1a,b). Based on this threshold, one section at 1 dpi and four sections at 2 dpi were excluded. After filtering, we normalized the number of detected genes across sections using a regularized negative binomial regression model (Hafemeister & Satija, 2019), thereby reducing technical variation while preserving biological heterogeneity (Fig. 2c,d). Following filtering and normalization, we detected 26 444 unique Arabidopsis genes in the RNA sequencing library of nematode‐infected Arabidopsis roots at 1 dpi, representing ±96% of the coding sequences in Arabidopsis. Additionally, we identified 2609 unique genes from *H. schachtii* at 1 dpi, representing ±10% of the nematode coding sequences. Likewise, at 2 dpi, we detected 26 175 unique Arabidopsis genes in the RNA sequencing library, representing ±95% of the coding sequences in Arabidopsis, and 10 655 nematode genes, representing ±40% of the nematode coding sequences. Thus, RNA tomography effectively captures a comprehensive representation of the Arabidopsis transcriptome and a representative portion of the *H. schachtii* transcriptome, particularly at later infection stages, confirming its sensitivity and robustness for gene expression analysis during infection.

**Fig. 3:**
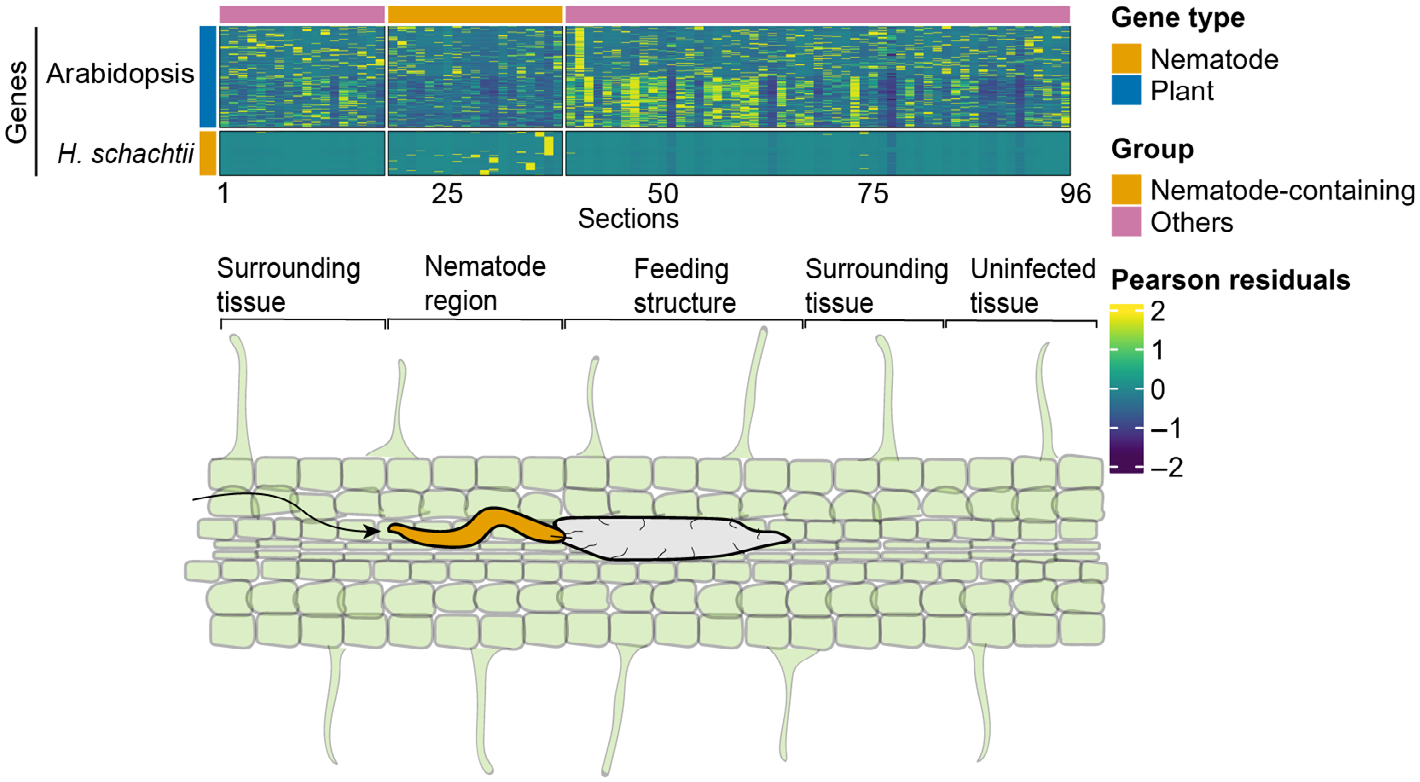
Schematic overview of RNA tomography on a cyst nematode‐infected *Arabidopsis thaliana* root. *Heterodera schachtii* (orange) migrates through the host root, as indicated by the black arrow, and transforms several host cells (green) into a feeding structure (gray). Feeding structure formation involves extensive transcriptional changes. Using RNA tomography, we can spatially map the expression of both Arabidopsis (blue) and *H. schachtii* (orange) genes across tissue sections. This enables us, for instance, to compare gene expression patterns within the feeding structure, surrounding plant tissue, and uninfected tissue farther away from the infection site, or to identify putative nematode effector genes based on similarity across expression patterns.

Next, we aimed to determine the position of the nematode within the samples by analyzing *H. schachtii* expression profiles. To establish a threshold, we plotted the number of *H. schachtii* genes per section in ascending order, following the same approach as described above, and set the threshold at 75 genes (Fig. S1c,d). Out of 95 sections, 13 sections contained at least 75 *H. schachtii* genes at 1 dpi (Fig. 2e). At 2 dpi, 21 out of 91 sections had > 75 nematode gene counts (Fig. 2f). However, in both samples, sections containing *H. schachtii* mRNA were not necessarily consecutive, even though we would expect that the nematode spans at least a few sections. Since nematode genes were detected in consecutive sections 57 to 68 at 1 dpi, and sections 20 to 39 at 2 dpi, we assume that these sections represent the nematode's position in the respective samples. We will refer to these sections as the ‘nematode region’. Altogether, these results demonstrate that RNA tomography can accurately capture the spatial distribution of *H. schachtii* genes within Arabidopsis roots, allowing us to presume the nematode's position (Fig. 3).

### The expression patterns of *H. schachtii* marker genes confirm the high resolution of RNA tomography maps

**Fig. 4:**
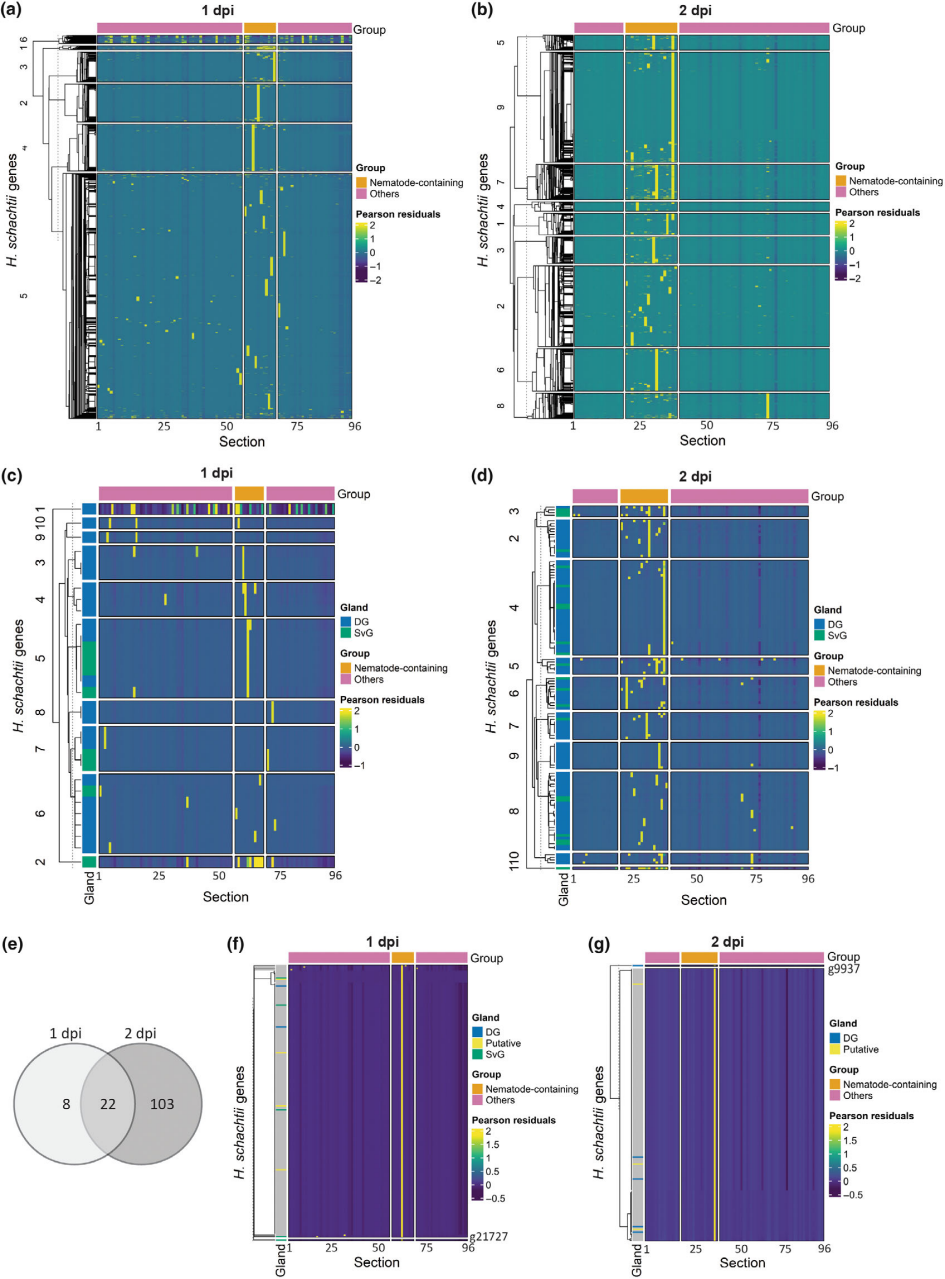
Revealing the expression patterns of *Heterodera schachtii* genes expressed in the dorsal and subventral glands to locate the nematode head within the *Arabidopsis thaliana* root and identify putative effector genes. This figure demonstrates the use of RNA tomography to locate the nematode head position and identify putative effector genes from spatial gene expression patterns of marker genes. (a, b) Heatmap showing the expression patterns of detected *H. schachtii* genes at 1 (a) and 2 d post inoculation (dpi) (b) on the *y*‐axis. The *x*‐axis represents 20 μm tissue sections, with consecutive sections containing > 75 *H. schachtii* genes, indicated in orange. Other sections with mostly plant genes and substantially less nematode mRNA are indicated in pink. The dendrogram represents the similarity among the selected *H. schachtii* genes based on Euclidean clustering. (c, d) Expression profiles of *H. schachtii* marker genes known to be expressed in either the dorsal gland (DG, blue) or subventral glands (SvG, green) across sections at 1 (c) and 2 dpi (d). (e) Venn diagram showing the number of *H. schachtii* genes known to be expressed in either the DG or SvG at 1 and/or 2 dpi. (f, g) The 200 *H. schachtii* genes exhibiting expression profiles most similar to subventral glands‐expressed gene 21 727 at 1 dpi (f) and dorsal gland gene 9937 at 2 dpi (g) based on Spearman correlation > 0.9. See Supporting Information Dataset S1 for the gene IDs displayed in plots (a–d, f, g).

To further study the nematode region, we examined whether spatial gene expression patterns could be observed within these sections. Hereto, we performed hierarchical clustering on normalized gene expression data and visualized the *H. schachtii* gene expression in a spatial gene expression map. The optimal number of clusters was determined using the second derivative of the WCSS (Fig. S2a–d). The heatmaps for both the 1 and 2 dpi samples indeed show that genes cluster together based on their spatial gene expression (Fig. 4a,b). For instance, at 1 dpi, clusters 2, 3, and 4 exhibited high gene expression in single sections throughout the nematode region. By contrast, genes in cluster 6 were also expressed outside the nematode region. Many of these genes have sequence similarity to Arabidopsis genes (Dataset S1), suggesting that these genes may be more conserved across species, like DNA replication, translation, or transcription. The spatial gene expression pattern at 2 dpi was even more distinct than at 1 dpi, with different clusters showing high gene expression in specific single sections within the nematode region, except for cluster 8, which shows high expression in section 74.

We hypothesized that gene clusters with high expression in specific sections might correspond to distinct anatomical structures in the nematode. To test this hypothesis, we first determined the optimal number of gene clusters (Fig. S3e–h) for visualizing the expression of genes known to be specifically expressed in either the dorsal or subventral glands, according to the recently updated *H. schachtii* effectorome (Molloy *et al*., 2024) (Fig. 4c,d). These pharyngeal glands secrete effector proteins essential for feeding structure initiation and development. At 1 dpi, we identified 30 annotated gland genes, including 22 expressed in the dorsal gland and 8 in the subventral glands. In the heatmap, seven clusters contained only dorsal gland‐expressed genes, most of which peaked in sections 60 and 61. Subventral glands‐expressed genes were predominantly highly expressed in section 62. At 2 dpi, the number of detected gland genes increased to 125, including 104 dorsal gland‐expressed genes and 21 subventral glands‐expressed genes. Compared to 1 dpi, the distinction between dorsal and subventral glands expression was less pronounced at 2 dpi. Three clusters contained only genes annotated as dorsal gland expressed. While most annotated genes were expressed in the nematode region, they peaked in different sections, with most genes highly expressed in section 38. Taken together, by mapping the spatial expression of annotated dorsal and subventral gland genes, we further validated our assumptions about the nematode's location within the samples and provided insight into the glands' location.

Considering that the timing of effector secretion is crucial during host invasion and the initiation of feeding structure formation, we determined whether the expression of these effectors is time‐dependent by comparing the genes expressed in the dorsal or subventral glands across both timepoints. Of the 30 genes expressed at 1 dpi, 22 were also expressed at 2 dpi, meaning that eight genes were specifically expressed at 1 dpi (Fig. 4e). Among the 125 annotated genes detected at 2 dpi, 103 genes were specifically expressed at 2 dpi. To further study the function of these genes, we used the *H. schachtii* effectorome (Molloy *et al*., 2024) and the eggnog‐mapper (v.2) (Cantalapiedra *et al*., 2021), a functional annotation tool for novel sequences (Dataset S2). This analysis revealed that the overlapping genes belong to various effector families, including the glutathione synthetase family, which plays an important role in regulating cellular redox homeostasis (Lilley *et al*., 2018), as well as cell‐wall degrading enzymes such as chitinase, cellulase, and pectate lyase.

The eight genes specifically expressed at 1 dpi include two genes encoding cellulose‐binding proteins, which support parasitism by reducing methyl esterified pectin in the cell wall (Hewezi *et al*., 2008), and an annexin. Genes specifically expressed at 2 dpi belong to effector families such as venom allergen‐like proteins, which suppress plant immunity (Lozano‐Torres *et al*., 2014), clavata3/embryo surrounding region (CLE)‐like peptides, which manipulate host developmental pathways to support feeding structure formation (Guo *et al*., 2017), and an expansin gene, which may regulate cell‐wall loosening during feeding structure formation (Fudali *et al*., 2008). Thus, our analysis reveals distinct temporal patterns of effector gene expression, suggesting that this expression is tightly regulated and that these effectors may play critical roles at different stages of parasitism.

Although many *H. schachtii* effectors are included in the effectorome, it is likely still incomplete (Molloy *et al*., 2024). Our spatial transcriptomics method enables the visualization of spatial gene expression, allowing us to identify genes with expression patterns similar to known marker genes for the subventral and dorsal glands, which may represent putative effectors. To identify putative effectors, we generated heatmaps displaying 200 genes similarly expressed as two marker genes: *H. schachtii* gene 21 727, a cellulase expressed in the subventral glands at 1 dpi in infective J2 nematodes (Siddique *et al*., 2022), and *H. schachtii* gene 9937, a glutathione synthase expressed in the dorsal gland of infective J2 nematodes at 2 dpi (Siddique *et al*., 2022). At 1 dpi, we identified several unannotated genes with expression patterns similar to g21727, including four putative effectors listed in the effectorome (Molloy *et al*., 2024), along with four genes expressed in the subventral glands and two dorsal gland‐expressed genes. Most of these genes were highly expressed in section 62, the same section as the marker gene (Fig. 4f). At 2 dpi, we identified 200 genes highly expressed in section 38, showing a similar pattern to the marker gene 9937 (Fig. 4g). Notably, four of the identified genes at 2 dpi were annotated as dorsal gland‐expressed genes (Siddique *et al*., 2022), and three were annotated as putative effectors in the effectorome, confirming the accuracy of our analysis. Thus, RNA tomography can identify putative effector genes based on their spatial expression patterns, offering a list of candidate genes for further investigation.

### Specific regions of the root exhibit distinct gene expression in response to nematode infection

**Fig. 5:**
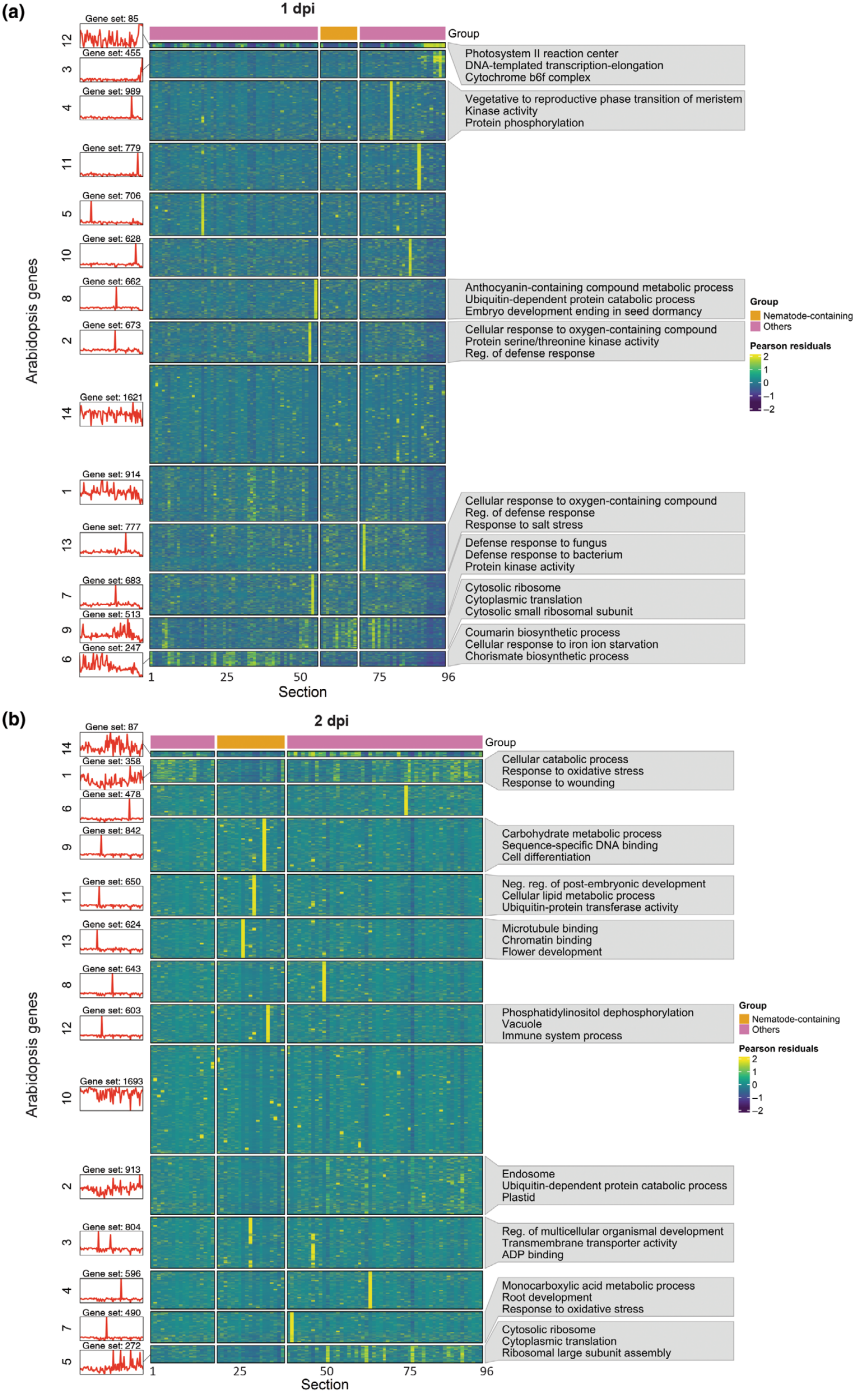
Heatmap revealing the expression patterns of the most variable *Arabidopsis thaliana* genes in response to *Heterodera schachtii* infection. The 10 000 most variably expressed genes across tissue sections were identified using the SCTransform package. The expression patterns of 9732 and 9053 Arabidopsis genes for 1 (a) and 2 d post inoculation (b), respectively, were visualized in the heatmap. The *y*‐axis represents Arabidopsis genes, while the *x*‐axis represents 20 μm tissue sections, with consecutive sections containing nematode mRNA indicated at the top of the heatmap in orange; feeding structures are initiated within or near these sections. Panels on the left show average gene expression patterns per cluster, while the top three Gene Ontology terms of clusters with possible roles in nematode infection cluster are displayed at the right side of the heatmap.

Our RNA tomography data has proven valuable in revealing the spatial gene expression patterns of the nematode within the host. Building on this, we used the data to study gene expression changes in the plant root during infection, as the molecular mechanisms underlying feeding structure development remain poorly understood. Specifically, we aimed to investigate how plant roots respond to nematode infection and identify the section(s) containing the nematode‐induced feeding structures. Since feeding structure formation occurs locally within the root, we first searched for up to 10 000 genes with the highest variability in expression across root sections, resulting in 9732 Arabidopsis genes at 1 dpi, and 9053 Arabidopsis genes at 2 dpi. We then determined the optimal number of clusters using the elbow method and its second derivative (Fig. S3a–d), performed hierarchical clustering on gene expression data, and visualized the gene expression patterns in a heatmap (Fig. 5a,b). To assess the biological relevance of these clusters, we performed Gene Ontology (GO) enrichment analyses, focusing on clusters with high or altered expression in or near the nematode region, where the feeding structure is expected to initiate and develop (Dataset S3).

At 1 dpi, we observed multiple clusters with high gene expression localized to a specific section. This aligns with our expectations as *H. schachtii* initiates feeding structures from a single, typically procambial or pericycle cell (Golinowski *et al*., 1996; Sobczak *et al*., 1997), which likely spans multiple sections. We focused on clusters 2, 7, and 8, as genes in these clusters were locally highly expressed near the nematode region, specifically near the head‐side of the nematode. The majority of enriched GO terms (FDR ≤ 0.05) associated with these clusters were related to plant defense. For instance, cluster 7 was enriched for ‘defense response to fungus’ (43 genes), ‘defense response to bacterium’ (63 genes), and ‘protein kinase activity’ (29 genes) (AbuQamar *et al*., 2008). Similarly, cluster 2 was enriched for ‘cellular response to oxygen‐containing compound’ (26 genes), ‘protein serine/threonine kinase activity’ (36 genes), and ‘regulation of defense response’ (29 genes).

Interestingly, genes in cluster 6 were highly expressed in sections located before the nematode's anterior region, but not in the nematode region or in neighboring sections at its posterior end. This cluster was enriched for genes involved in the biosynthesis of compounds that may play a role in plant defense, including genes associated with the ‘coumarin biosynthetic process’ (five genes), a family of plant secondary metabolites (Vismans *et al*., 2022), and the ‘chorismate biosynthetic processes’ (five genes), a precursor for salicylic acid biosynthesis (Tzin & Galili, 2010). A similar expression pattern was observed at 2 dpi, where cluster 1 showed gene expression on both sides of the nematode region, though expression was dampened within the nematode region and its immediate neighboring sections. This cluster was enriched for genes related to stress responses, such as ‘cellular catabolic process’ (23 genes), ‘response to oxidative stress’ (20 genes), and ‘response to wounding’ (34 genes). These spatially distinct expression patterns suggest a localized regulation of defense‐related gene expression during early stages of nematode infection.

At 2 dpi, genes in clusters 3, 9, 11, 12, and 13 peaked in a specific section of the nematode region, located close to each other. We speculate that this area likely corresponds to the feeding structure. Significant GO terms for these clusters include ‘cell differentiation’ (25 genes), suggesting that the affected cells are transformed into specialized feeding structures, ‘transmembrane transporter activity’ (19 genes), likely involved in nutrient supply, and ‘vacuole’ (11 genes), as in feeding structures, the large central vacuole is replaced by numerous vesicles of various sizes (Golinowski *et al*., 1996; Baranowski *et al*., 2018). Notably, genes in cluster 13 were also enriched for ‘flower development’ (12 genes), while we only captured root tissue. However, in flowering plants, three cell fusion events occur within the embryo sac. One sperm cell fuses with the egg cell to form the diploid zygote, while a second sperm cell fuses with the central cell to form the endosperm (Sprunck & Dresselhaus, 2015). After fertilization of the central cell, the persistent synergid fuses with the endosperm (Maruyama *et al*., 2015). Since cell fusion is a unique event that only occurs in the embryo sac within the developing ovule, we suggest that cyst nematodes may hijack components of flower development for the fusion of cells during feeding structure formation.

Altogether, our GO term enrichment analysis confirms that the plant responds to nematode infection, and its response changes over time. At 1 dpi, we observe plant defense responses, while at 2 dpi, in more developed feeding structures, we might have identified the initiation of a feeding structure. However, further research is necessary to validate the events leading to nematode‐induced feeding structure initiation and development.

## Discussion

Plant cells coordinate both developmental processes and responses to environmental stimuli. Yet, current methodologies for studying gene expression either lack spatial resolution, have low throughput, cover limited tissue areas, are expensive, or require prior knowledge to understand these complex responses. To address this, we applied RNA tomography, a spatial transcriptomics technology, in Arabidopsis roots infected with the cyst nematode *H. schachtii*. This plant‐nematode combination features asynchrony of infection and localized cellular changes during feeding structure formation, demanding high‐resolution spatio‐temporal gene expression analysis. We used RNA tomography to spatially map gene expression in Arabidopsis roots and nematodes, shedding light on the molecular mechanisms underlying the initiation of feeding structures induced by cyst nematodes.

We detected *c*. 96 and 95% of Arabidopsis' coding genes at 1 and 2 dpi, respectively. These percentages are comparable to those in single‐cell RNA‐seq datasets (*c.* 90.00%) (Denyer *et al*., 2019; Shahan *et al*., 2022) and bulk RNA‐seq (*c.* 82.64%) (Siddique *et al*., 2022), demonstrating that RNA tomography is highly sensitive and feasible for use in plants. Furthermore, we detected *c*. 10% and 40% of *H. schachtii*'s coding genes at 1 and 2 dpi, respectively. The lower number of detected nematode genes is likely due to low input material, the focus on the feeding structure, which may not necessarily include the entire nematode in the sample, variations in the nematode's position within the samples; a strict time‐dependent gene regulation; or a combination of these factors.

We confirmed localized gene expression, mapped known dorsal and subventral gland‐expressed genes of *H. schachtii* (Molloy *et al*., 2024) and identified 200 *H. schachtii* genes with similar expression patterns. The interpretation of localized gene expression may be affected by the one‐dimensional nature of our data, which compresses heterogeneity in two and three dimensions and can lead to tissue overlap in sections, as nematode geometries can be serpentine. Remarkably, mRNA of some *H. schachtii* genes were also found outside the nematode region. This signal, unlikely originating from measurement noise, suggests potential mechanisms for nematode manipulation of the host. For example, *H. schachtii* secretes CLE‐like peptides that mimic Arabidopsis CLEs, hijacking the plant's developmental signaling pathways to reprogram root cells and promote feeding structure formation (Wang *et al*., 2010). Nematode CLE‐like effectors are processed by the host's endoplasmic reticulum secretory pathway, including post‐translational modifications, and are delivered to the extracellular space of nematode‐infected host cells, where they can be recognized by plant receptors (Wang *et al*., 2021). This re‐trafficking may allow the nematode to communicate with cells adjacent to the initial feeding cell, facilitating the expansion of the syncytium. Alternatively, *H. schachtii* may secrete mRNA molecules, similar to animal parasitic nematodes (Buck *et al*., 2014; Taylor *et al*., 2020), and use the host machinery for translation at certain locations in the host to facilitate the infection process. Plants also use mRNA for long‐distance communication (Kitagawa *et al*., 2024), *H. schachtii* might make use of this process for infection. Secretion of mRNA could reduce the energetic costs for *H. schachtii* while increases its reach within the host.

Several plant gene clusters showed spatially restricted expression. One gene cluster, highly expressed outside the nematode region but showing reduced expression within this region, was enriched, for defense‐related processes, including coumarin and chorismate biosynthesis at 1 dpi. Both, the accumulation of the coumarin scopoline (Beesley *et al*., 2023), and of salicylic acid, synthesized from chorismate (Tzin & Galili, 2010), can alter nematode susceptibility (Zinovieva *et al*., 2011). This spatial expression pattern suggests that the nematode may actively suppress or evade localized host defense responses during early infection stages, highlighting the dynamic and complex interplay of plant‐nematode interactions. Whether these gene expression patterns result in feeding structures that promote female or male development remains unknown, but analyzing multiple individual samples could reveal patterns associated with sexual dimorphism in cyst nematodes.

Unexpectedly, at 2 dpi, one cluster was enriched for flower development, suggesting potential similarities between gynoecium development and early feeding structure formation. Both processes involve cell fusion (Maruyama *et al*., 2015), plant hormonal reprogramming, for instance of auxin and cytokinin (Goverse & Bird, 2011; Marsch‐Martínez & de Folter, 2016), and share some key developmental regulators (Moubayidin & Østergaard, 2017; Correa *et al*., 2018). These findings highlight how RNA tomography can reveal gene expression patterns and help identify promising candidate gene lists for further in‐depth research.

RNA tomography offers advantages for plant studies over other spatial transcriptomics technologies, including affordability, accessibility, that is requiring standard laboratory equipment (Junker *et al*., 2014; Holler & Junker, 2019), and the ability to analyze large tissue areas with the possibility of reaching fully untargeted 3D RNA positioning (Junker *et al*., 2014; Mayeur *et al*., 2021). We foresee that the lack of single‐cell resolution and limitations inherent to short‐read sequencing in RNA tomography studies will be overcome either computationally by integrating with single‐cell data or by the rapid advances in long‐read sequencing technologies.

In summary, our findings provide novel insights into the gene expression in and around feeding structures induced by cyst nematodes. We demonstrate that RNA tomography allows the transcriptome‐wide analysis of both host and pathogen spatiotemporally, enabling the identification of regions of interest, the localization of gene expression using marker genes, and candidate gene selection based on expression profiles. We anticipate RNA tomography to have broad applicability across various plant studies involving development, biotic, or abiotic stresses, and its potential integration with existing and future single‐cell sequencing.

## Competing interests

None declared.

## Author contributions

JLL‐T and HCK: conceptualization; YW, SW, KA and AP: data analysis; JLL‐T: funding acquisition; JM, J‐JW and AP: methodology; AP: investigation; YW and AP: visualization; AP: writing – original draft; YW, J‐JW, JM, SW, GS, HCK and JLL‐T: writing – review and editing. AP and YW contributed equally to this work.

## Disclaimer

The New Phytologist Foundation remains neutral with regard to jurisdictional claims in maps and in any institutional affiliations.

## Supporting information


**Dataset S1** Protein sequence similarity between *Heterodera schachtii* genes expressed outside the nematode region to Arabidopsis proteins.

**Dataset S2** Detected *Heterodera schachtii* genes, annotated gland genes and genes most similarly expressed as dorsal (DG9937) and subventral glands (SvG21727) marker genes.

**Dataset S3** Most variably expressed Arabidopsis genes and corresponding Gene Ontology terms, 1‐ and 2‐d post inoculation with *Heterodera schachtii*.

**Fig. S1** Determining the threshold for filtering low‐count sections based on gene counts.
**Fig. S2** Identification of the optimal number of clusters for *Heterodera schachtii* spatial gene expression maps using the elbow method and its second derivative.
**Fig. S3** Identification of the optimal number of clusters for Arabidopsis spatial gene expression maps using the elbow method and its second derivative.
Please note: Wiley is not responsible for the content or functionality of any Supporting Information supplied by the authors. Any queries (other than missing material) should be directed to the *New Phytologist* Central Office.

## Data Availability

Raw sequencing data from this article can be found in the GEO database under accession number GSE297648. The processed UMI matrixes and source code for customized plots are available at: https://github.com/wwinnerhoo/Tomo‐seq‐for‐Arab‐Hsch.git.
