# Supplementary material for: RNA tomography reveals spatial gene expression maps of Arabidopsis thaliana roots infected with Heterodera schachtii
Source: New Phytol. 2025 Oct 17;249(1):588–602. doi: 10.1111/nph.70674 (PMC12676076; doi:10.1111/nph.70674)
Supplement: Supplementary file 4 — Fig. S1 Determining the threshold for filtering low‐count sections based on gene counts. Fig. S2 Identification of the optimal number of clusters for Heterodera schachtii spatial gene expression maps using the elbow method and its second derivative. Fig. S3 Identification of the optimal number of clusters for Arabidopsis spatial gene expression maps using the elbow method and its second derivative. Please note: Wiley is not responsible for the content or functionality of any Supporting Information supplied by the authors. Any queries (other than missing material) should be directed to the New Phytologist Central Office. [file NPH-249-588-s003.pdf]

New Phytologist

***New Phytologist* Supporting Information**

Article title: RNA tomography reveals spatial gene expression maps of *Arabidopsis thaliana* roots infected with *Heterodera schachtii*

Authors: Anna Pijnacker*, Yuhao Wang*, Jaap-Jan Willig, Jonas Mars, Steffen Werner, Kelvin Adema, Geert Smant, Hendrik C. Korswagen, Jose L. Lozano-Torres

Article acceptance date: 27 September 2025

The following Supporting Information is available for this article:

Figure S1. Determining the threshold for filtering low-count sections based on gene counts.

Figure S2. Identification of the optimal number of clusters for *Heterodera schachtii* spatial gene expression maps using the elbow method and its second derivative.

Figure S3. Identification of the optimal number of clusters for Arabidopsis spatial gene expression maps using the elbow method and its second derivative.

Dataset S1. Protein sequence similarity between *Heterodera schachtii* genes expressed outside the nematode region and Arabidopsis proteins.

Dataset S2. Detected *Heterodera schachtii* genes, annotated gland genes and genes most similarly expressed as dorsal (DG9937) and subventral glands (SvG21727) marker genes.

Dataset S3. Most variably expressed Arabidopsis genes and corresponding Gene Ontology terms, 1- and 2-days post inoculation with *Heterodera schachtii*.

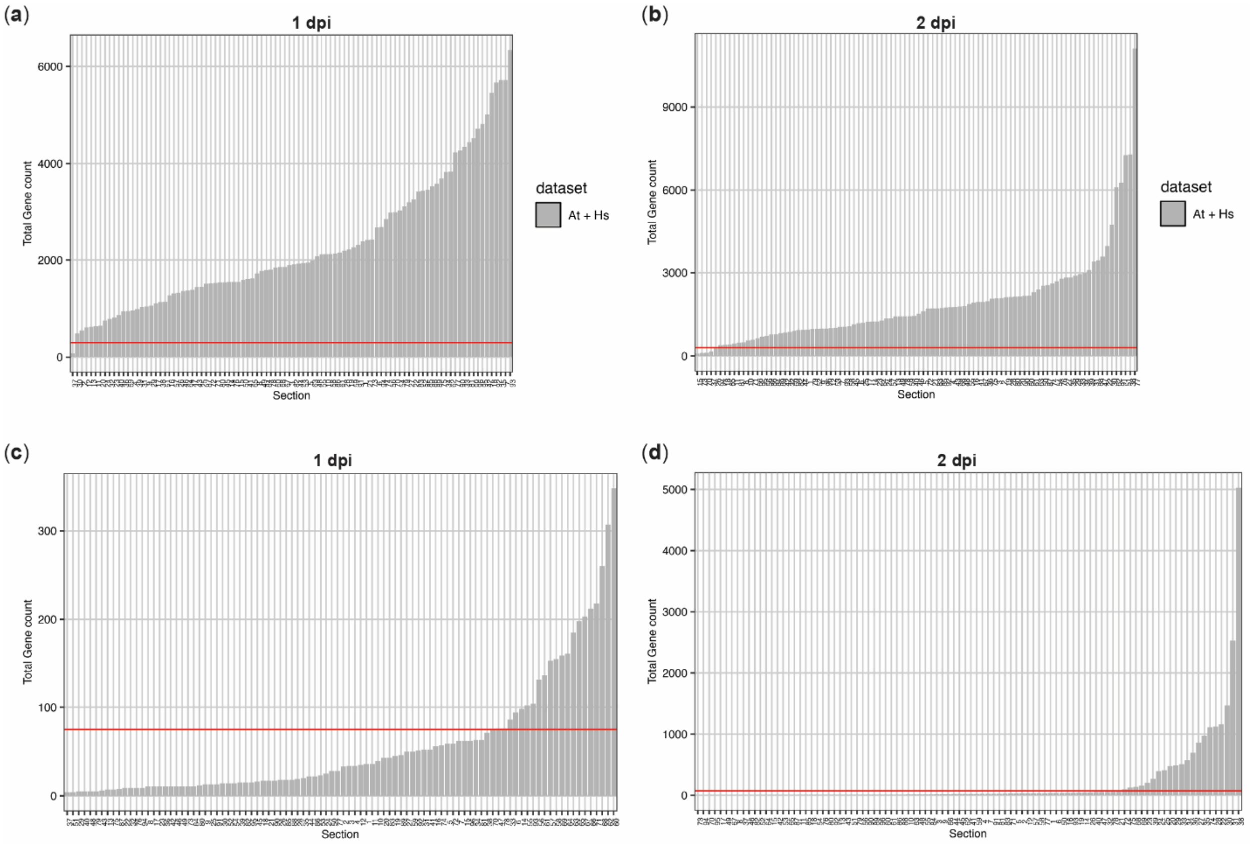

***Fig. S1.* Determining the threshold for filtering low-count sections based on the detected number of genes.** This figure illustrates how thresholds for filtering sections with low numbers of *Arabidopsis thaliana* and *Heterodera schachtii* genes were determined. (**a,b**) The number of detected Arabidopsis and *H. schachtii* genes per section was summed and plotted in ascending order for the 1 (**a**) and 2 days post inoculation (**b**) samples. Arabidopsis gene counts are shown in light grey, while *H. schachtii* counts are represented in dark grey. The threshold for filtering, set at 300 total gene counts, was determined by identifying the largest discontinuity between consecutive values in the cumulative distribution. This discontinuity indicates the superposition of two distinct behavioral regimes, low-quality and high-quality, and is indicated by the red line. (**c,d**) To determine a threshold for selecting the sections containing nematode mRNA, the number of *H. schachtii* genes per section was plotted in ascending order for the 1 dpi (**c**) and 2 dpi (**d**) samples. The red line indicates the threshold, defined by the largest discontinuity between consecutive values, corresponding to 75 *H. schachtii* genes. This figure supports **Fig. 2**.

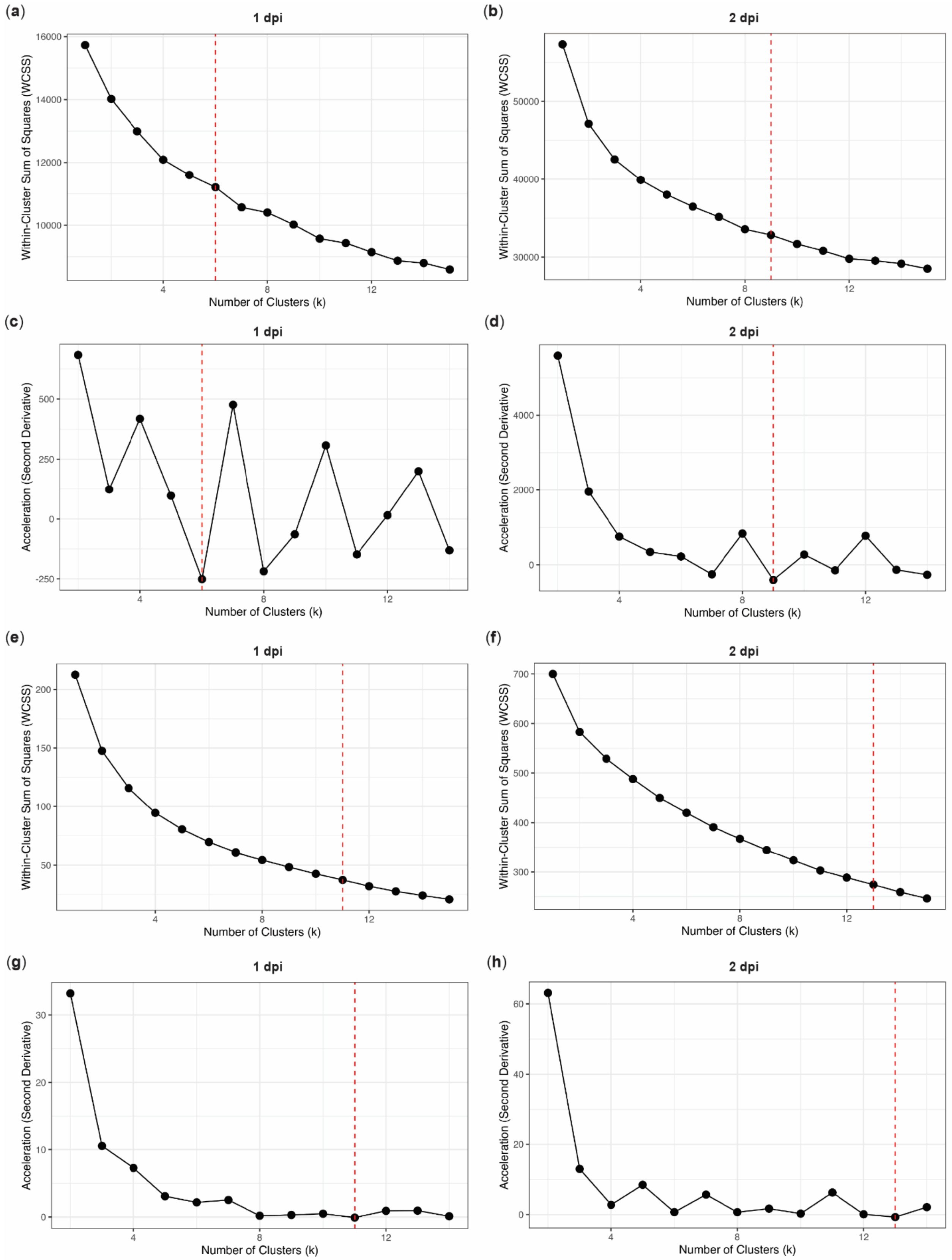

Fig. S2. Identification of the optimal number of clusters for *Heterodera schachtii* spatial gene

expression maps using the elbow method and its second derivative. The x-axis represents the number of clusters, ranging from 1 to 15, while the y-axis shows the within-cluster sum of squares (WCSS). The dashed red line indicates the optimal number of clusters for the respective sample. (**a,b**) Elbow plots for all detected *H. schachtii* genes in the 1 (**a**) and 2 days post inoculation (**b**) samples. (**c,d**) The second derivative of the WCSS plotted on the y-axis identifies the optimal number of clusters for the 1 (**c**) and 2 dpi (**d**) samples based on the point of maximum curvature. (**e,f**) Elbow plots for detected *H. schachtii* genes known to be expressed in the dorsal or subventral glands for the 1 (**e**) and 2 dpi (**f**) samples. (**g,h**) The point of maximum curvature for the 1 (**g**) and 2 dpi (**h**) samples, determined by plotting the second derivative of WCSS. This figure supports **Fig. 3**.

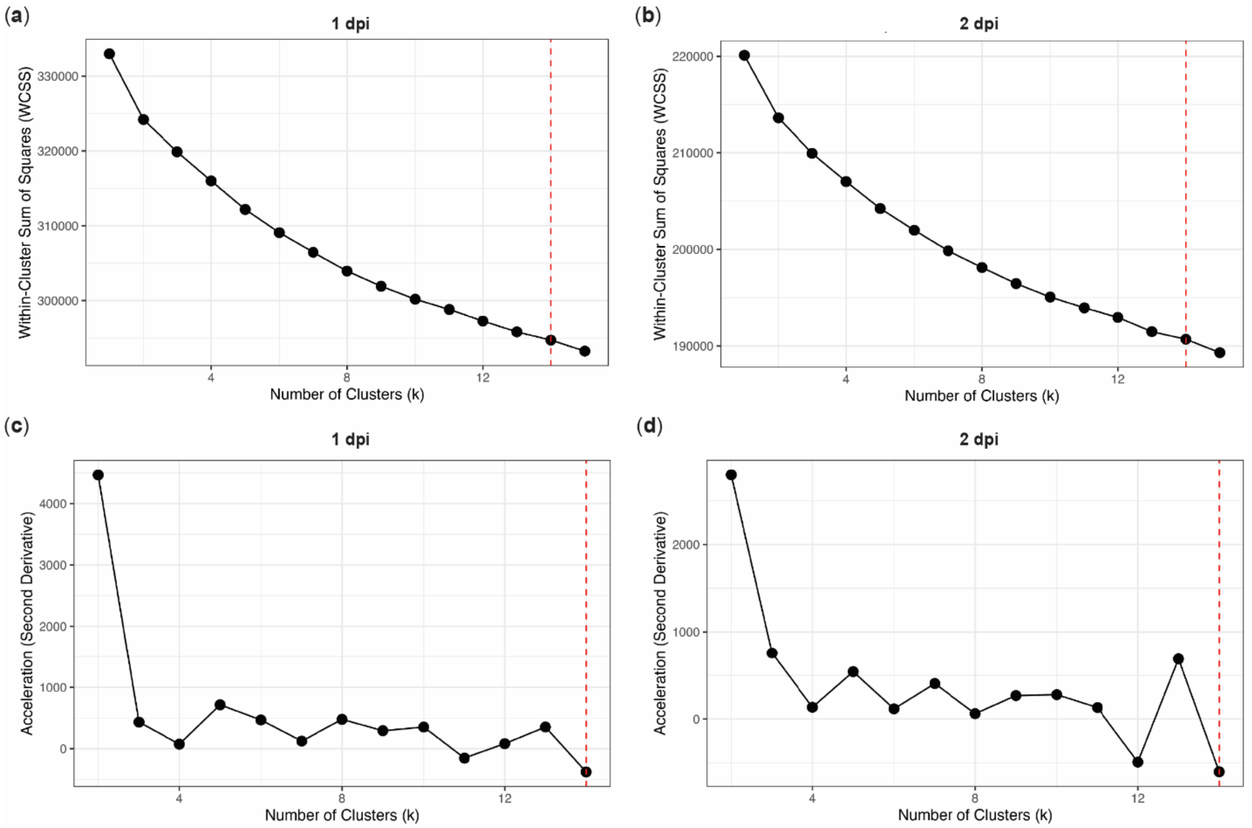

Fig. S3. **Identification of the optimal number of clusters for *Arabidopsis thaliana* spatial gene expression maps using the elbow method and its second derivative.** The x-axis represents the number of clusters, ranging from 1 to 15, while the y-axis shows the within-cluster sum of squares (WCSS). The dashed red line indicates the optimal number of clusters for the respective sample. (**a,b**) Elbow plots for the 9,732 and 9,937 most variably expressed Arabidopsis genes across sections at 1 (**a**) and 2 days post inoculation (**b**), respectively. (**c,d**) The second derivative of the WCSS plotted on the y-axis identifies the optimal number of clusters for the 1 (**c**) and 2 dpi (**d**) samples based on the point of maximum curvature. This figure supports **Fig. 4**.

References

Molloy, B., Shin, D. S., Long, J., Pellegrin, C., Senatori, B., Vieira, P., ... & Eves-van den Akker, S. (2024). The origin, deployment, and evolution of a plant-parasitic nematode effectorome. PLoS pathogens, 20(7), e1012395.
